# Bioactive Natural Products from the Red Sea

**DOI:** 10.3390/md19060289

**Published:** 2021-05-21

**Authors:** Mostafa E. Rateb, Usama Ramadan Abdelmohsen

**Affiliations:** 1 School of Computing, Engineering & Physical Sciences, University of the West of Scotland, Paisley PA1 2BE, UK; 2 Department of Pharmacognosy, Faculty of Pharmacy, Deraya University, New Minia 61111, Egypt; 3 Egypt 11 Department of Pharmacognosy, Faculty of Pharmacy, Minia University, Minia 61519, Egypt

The marine environment has proven to be a rich source of diverse natural products with relevant activities such as anticancer, anti-inflammatory, antiepileptic, immunomodulatory, antifungal, antiviral, and antiparasitic [1,2]. The global marine pharmaceutical clinical pipeline comprises of 48 compounds originating from different marine invertebrates and marine microorganisms, including 15 approved drugs by the most representative approving agencies, 5 drug candidates in phase III, 12 in phase II, and 16 in phase I of drug development clinical phases [3]. Marine invertebrates and associated microorganisms are capable of synthesizing diverse classes of secondary metabolites and, in some cases, novel chemical leads of which terrestrial counterparts have never been discovered.

The Red Sea is the world’s northernmost tropical sea, acting as an inlet of the Indian Ocean, lying between Africa and Asia. It has a surface area of roughly 450,000 km^2^ and is approximately 2250 km long, with a maximum depth of around 3000 m. The Red Sea is approximately 5% greater than the world average salinity, due to a high rate of evaporation and a lack of significant rivers or streams draining into it [4]. The Red Sea is a rich and diverse ecosystem due to the 2000 km of coral reef extending along its coastline. It is inhabited by over 1000 invertebrate species and 200 soft and hard corals [5]. Due to this high biodiversity and limited research, the Red Sea is a promising, underexplored habitat for the discovery of new bioactive marine natural products.

This Special Issue contains nine articles, including eight research articles on different topics related to the natural products derived from the Red Sea and one review article. In the following sections, we would provide a short overview of what the reader will find in our Special Issue.

Qader et al. reported the isolation and identification of a new cyclic tripeptide named epicotripeptin, along with four known cyclic dipeptides and one acetamide, derivative from seagrass-associated endophytic fungus *Epicoccum nigrum* M13 recovered from the Red Sea. Moreover, they reported two new compounds, cyclodidepsipeptide phragamide A and trioxobutanamide derivative phragamide B, together with eight known compounds from plant-derived endophyte *Alternaria alternata* 13A collected from a saline lake of the Wadi El Natrun depression in the Sahara Desert. The antimicrobial screening indicated that seven of the tested compounds exhibited considerable (MIC range of 2.5–5 µg/mL) to moderate (10–20 µg/mL) antibacterial effect against the tested Gram-positive strains, and moderate to weak (10–30 µg/mL) antibacterial effect against Gram-negative strains. On the other hand, four of the tested compounds showed considerable antibiofilm effects against biofilm-forming Gram-positive and Gram-negative strains [6].

Cocultivation is a productive technique to trigger microbes’ biosynthetic capacity by mimicking the natural habitats’ features, principally by competition for food and space and interspecies cross-talks. Alhadrami et al. used coculture of two Red Sea-derived actinobacteria, *Actinokineospora spheciospongiae* strain EG49 and *Rhodococcus* sp. UR59, resulting in the induction of several non-traced metabolites in their axenic cultures, which were detected using LC–HRMS metabolomics analysis. Antimalarial guided isolation of the cocultured fermentation led to the isolation of the angucyclines actinosporins E, H, G, tetragulol, and the anthraquinone capillasterquinone B. The isolated angucycline and anthraquinone compounds exhibited in vitro antimalarial activity and good biding affinity against lysyl-tRNA synthetase (PfKRS1), highlighting their potential to be developed as a new antimalarial structural motif [7].

Tammam et al. were able to isolate six new, and twenty known, steroids from the extract of the soft coral *Sinularia polydactyla*, collected from the Hurghada reef in the Red Sea. They evaluated the cytotoxic, anti-inflammatory, anti-angiogenic, and neuroprotective activities of the isolated compounds and their effect on androgen receptor-regulated transcription in human tumours and non-cancerous cells. Two steroids showed significant cytotoxicity in the low micromolar range against the HeLa and MCF7 cancer cell lines. Additionally, two compounds exhibited neuroprotective activity on neuron-like SH-SY5Y cells [8].

Shaala et al. investigated the actinomycete strain *Streptomyces coelicolor* LY001 recovered from the sponge *Callyspongia siphonella*. The chemical analysis resulted in the isolation of three new natural chlorinated 3-phenylpropanoic acid derivatives, 3-(3,5-dichloro-4-hydroxyphenyl)propanoic acid, 3-(3,5-dichloro-4-hydroxyphenyl)propanoic acid methyl ester, and 3-(3-chloro-4-hydroxyphenyl)propanoic acid, along with 3-phenylpropanoic acid, E-cinnamic acid, and the diketopiperazine alkaloids cyclo(l-Phe-trans-4-OH-l-Pro) and cyclo(l-Phe-cis-4-OH-d-Pro). The compounds demonstrated significant and selective activities towards *Escherichia coli*, *Staphylococcus aureus* and *Candida albicans* [9].

In the article by Abdelhameed et al., the seagrass *Thalassodendron ciliatum* (Forssk.) Den Hartog was investigated. In this work, a new ergosterol derivative named thalassosterol was identified from the methanolic extract of *T. ciliatum*, along with two known sterols, namely ergosterol and stigmasterol. Thalassosterol exhibited significant in vitro antiproliferative potential against the human cervical cancer cell line (HeLa), and human breast cancer (MCF-7) cell lines. Those results aligned with docking studies on the new sterol which explained the possible binding interactions with an aromatase enzyme; this inhibition is beneficial in both cervical and breast cancer therapy [10].

Cocultivation has been known as an effective approach for the enhancement of the production of natural products from bacteria and fungi. In the study by Hifnawy et al. liquid chromatography coupled with high-resolution mass spectrometry-assisted metabolomic profiling of two sponge-associated actinomycetes, *Micromonospora* sp. UR56 and *Actinokineospora* sp. EG49, resulted in the induction of phenazine-derived compounds that were later identified, upon fermentation, as dimethyl phenazine-1,6-dicarboxylate, phenazine-1,6-dicarboxylic acid mono methyl ester (phencomycin, phenazine-1-carboxylic acid (tubermycin), *N*-(2-hydroxyphenyl)-acetamide, and p-anisamide. The antibacterial, antibiofilm, and cytotoxic properties of these metabolites were evaluated via in vitro and docking studies. This study highlighted that microbial cocultivation is an efficient tool for the discovery of new antimicrobial candidates and indicated phenazines as potential lead compounds for further development as antibiotic scaffolds [11].

Abdelhameed et al. reported the isolation of two new compounds: a ceramide, stylissamide A, and a cerebroside, stylissoside A, from the Red Sea sponge *Stylissa carteri*. Metabolomic profiling revealed the presence of diverse secondary metabolites, mainly oleanane-type saponins, phenolic diterpenes, and lupane triterpenes. They also investigated the in vitro cytotoxic activity of the isolated compounds against two human cancer cell lines, MCF-7 and HepG2. Molecular docking experiments showed that both compounds displayed high affinity to the SET protein and inhibitor 2 of protein phosphatase 2A (I2PP2A), a possible mechanism for their cytotoxic activity [12].

El-Kashef et al. investigated the marine-derived fungus *Aspergillus falconensis*, cultivated from sediment collected from the Canyon at Dahab, Red Sea, and reported a yield of two new chlorinated azaphilones, falconensins O and P in addition to four known azaphilone derivatives. Interestingly, replacing NaCl with NaBr induced the accumulation of three additional new azaphilones, falconensins Q−S, including two brominated derivatives along with three known analogues. Some of the tested compounds showed NF-κB inhibitory activity against the triple-negative breast cancer cell line MDA-MB-231. This study highlights the OSMAC approach as a successful approach to increase the diversity of secondary metabolites from marine fungi [13].

El-Hossary et al. provided a comprehensive review that compares the natural products recovered from the Red Sea in terms of ecological role and pharmacological activities. In this review, which covers the literature to the end of 2019, they summarized the diversity of bioactive secondary metabolites derived from Red Sea micro- and macro-organisms, and discuss their biological potential whenever applicable. Moreover, the diversity of the Red Sea organisms is highlighted, as well as genomic potential [14].

Finally, we could see that Red Sea derived natural products were diverse in terms of chemical scaffolds as well as pharmacological activities.

As guest editors, we appreciate the efforts provided by all of the authors who contributed their excellent results to this Special Issue, all of the reviewers who carefully evaluated the submitted manuscripts, and the editorial boards of Marine Drugs for their support and kind help.

## References

[1] Carroll A.R., Copp B.R., Davis R.A., Keyzers R.A., Prinsep M.R. (2020). Marine natural products. Nat. Prod. Rep..

[2] Carroll A.R., Copp B.R., Davis R.A., Keyzers R.A., Prinsep M.R. (2019). Marine natural products. Nat. Prod. Rep..

[3] https://www.marinepharmacology.org/.

[4] https://www.worldatlas.com/seas/red-sea.html.

[5] https://www.newworldencyclopedia.org/entry/Red_Sea.

[6] Qader M.M., Hamed A.A., Soldatou S., Abdelraof M., Elawady M.E., Hassane A.S.I., Belbahri L., Ebel R., Rateb M.E. (2021). Antimicrobial and Antibiofilm Activities of the Fungal Metabolites Isolated from the Marine Endophytes *Epicoccum nigrum* M13 and *Alternaria alternata* 13A. Mar. Drugs.

[7] Alhadrami H.A., Thissera B., Hassan M.H.A., Behery F.A., Ngwa C.-J., Hassan H.M., Pradel G., Abdelmohsen U.R., Rateb M.E. (2021). Bio-Guided Isolation of Antimalarial Metabolites from the Coculture of Two Red Sea Sponge-Derived *Actinokineospora* and *Rhodococcus* spp. Mar. Drugs.

[8] Tammam M.A., Rárová L., Kvasnicová M., Gonzalez G., Emam A.M., Mahdy A., Strnad M., Ioannou E., Roussis V. (2020). Bioactive Steroids from the Red Sea Soft Coral *Sinularia polydactyla*. Mar. Drugs.

[9] Shaala L.A., Youssef D.T.A., Alzughaibi T.A., Elhady S.S. (2020). Antimicrobial Chlorinated 3-Phenylpropanoic Acid Derivatives from the Red Sea Marine Actinomycete *Streptomyces coelicolor* LY001. Mar. Drugs.

[10] Abdelhameed R.F.A., Habib E.S., Goda M.S., Fahim J.R., Hassanean H.A., Eltamany E.E., Ibrahim A.K., AboulMagd A.M., Fayez S., Abd El-kader A.M. (2020). Thalassosterol, a New Cytotoxic Aromatase Inhibitor Ergosterol Derivative from the Red Sea Seagrass *Thalassodendron ciliatum*. Mar. Drugs.

[11] Hifnawy M.S., Hassan H.M., Mohammed R., Fouda M.M., Sayed A.M., Hamed A.A., AbouZid S.F., Rateb M.E., Alhadrami H.A., Abdelmohsen U.R. (2020). Induction of Antibacterial Metabolites by Co-Cultivation of Two Red-Sea-Sponge-Associated Actinomycetes *Micromonospora* sp. UR56 and *Actinokinespora* sp. EG49. Mar. Drugs.

[12] Abdelhameed R.F.A., Habib E.S., Eltahawy N.A., Hassanean H.A., Ibrahim A.K., Mohammed A.F., Fayez S., Hayallah A.M., Yamada K., Behery F.A. (2020). New Cytotoxic Natural Products from the Red Sea Sponge *Stylissa carteri*. Mar. Drugs.

[13] El-Kashef D.H., Youssef F.S., Hartmann R., Knedel T., Janiak C., Lin W., Reimche I., Teusch N., Zhen Liu Z., Proksch P. (2020). Azaphilones from the Red Sea Fungus *Aspergillus falconensis*. Mar. Drugs.

[14] El-Hossary E.M., Abdel-Halim M., Ibrahim E.S., Pimentel-Elardo S.M., Nodwell J.R., Handoussa H., Abdelwahab M.F., Holzgrabe U., Abdelmohsen U.R. (2020). Natural Products Repertoire of the Red Sea. Mar. Drugs.

